# Diagnostic utility of FAPI PET/CT in radioiodine-refractory thyroid cancer and TENIS syndrome: a systematic review

**DOI:** 10.3389/or.2026.1811291

**Published:** 2026-06-03

**Authors:** Akram Al-Ibraheem, Saad Ruzzeh, Ahmad Al-Muhtaseb, Ahmed Saad Abdlkadir, Serin Moghrabi, Hongcheng Shi, Habibollah Dadgar, Mahed Al-Foqahaa, Marwah Abdulrahman, Soud Al-Qasem, Issa Mohamad, Gerasimos P. Sykiotis

**Affiliations:** 1 Department of Nuclear Medicine, King Hussein Cancer Center (KHCC), Amman, Jordan; 2 School of Medicine, University of Jordan, Amman, Jordan; 3 Department of Radiology, Istishari Hospital, Amman, Jordan; 4 Department of Nuclear Medicine, Baghdad Radiotherapy and Nuclear Medicine Hospital, Bab Al-Muadham, Baghdad, Iraq; 5 Department of Nuclear Medicine, Zhongshan Hospital, Fudan University, Shanghai, China; 6 Nuclear Medicine Research Center, Mashhad University of Medical Sciences, Mashhad, Iran; 7 Department of Radiation Oncology, King Hussein Cancer Center (KHCC), Amman, Jordan; 8 Service of Endocrinology, Diabetology and Metabolism, Lausanne University Hospital and University of Lausanne, Lausanne, Switzerland

**Keywords:** differentiated thyroid carcinoma, FAPI PET/CT, FDG PET/CT, molecular imaging, RAIR, TENIS syndrome

## Abstract

**Background:**

Fibroblast activation protein inhibitor (FAPI) positron emission tomography/computed tomography (PET/CT) has emerged as a promising molecular imaging modality with favorable lesion-to-background contrast across several malignancies. In radioiodine-refractory thyroid carcinoma (RAIR-TC) and thyroglobulin-elevated negative iodine scintigraphy (TENIS) syndrome, conventional radioiodine imaging and fluorodeoxyglucose (FDG) PET/CT may be limited in selected clinical scenarios. This systematic review evaluated the diagnostic utility, comparative performance, and potential clinical impact of FAPI PET/CT in this patient population.

**Methods:**

PubMed, Embase, and Scopus were searched from inception to 6 August 2025 according to PRISMA 2020 guidelines. Eligible studies included original clinical studies and case reports evaluating FAPI PET/CT in RAIR-TC or TENIS syndrome. Non-original, non-human, *in vitro*, conference abstract, and non-English publications were excluded. Two reviewers independently performed study selection, data extraction, and quality assessment. QUADAS-2 was applied to eligible cohort studies.

**Results:**

Eight studies comprising 167 patients were included: three prospective studies, one retrospective study, and four case reports. FAPI PET/CT showed promising complementary diagnostic value in selected clinical scenarios, particularly for lesions with low FDG conspicuity or high physiologic FDG background. In one prospective cohort of 24 patients, [^68^Ga]Ga-DOTA-FAPI-04 PET/CT detected disease in 87.5% of patients. In the largest retrospective cohort (n = 117), [^68^Ga]Ga-DOTA.SA.FAPi showed higher detection than FDG PET/CT for selected metastatic sites, including lymph nodes, liver, and brain. However, comparative performance varied across tracers, lesion sites, and study designs; one prospective [^18^F]FAPI-74 study reported lower lesion detection than FDG PET/CT.

**Conclusion:**

FAPI PET/CT demonstrates promising but preliminary diagnostic utility in RAIR-TC and TENIS syndrome. Current evidence supports a complementary role in selected patients, but remains insufficient to establish routine diagnostic superiority over FDG PET/CT. Larger prospective multicenter studies with standardized protocols and robust reference validation are required.

**Systematic Review Registration:**

https://www.crd.york.ac.uk/PROSPERO/view/CRD420251123809.

## 1 Introduction

Differentiated thyroid carcinoma (DTC) is the most common endocrine malignancy worldwide, with a steadily increasing incidence over recent decades. It comprises papillary, follicular, and oncocytic thyroid carcinoma (PTC, FTC, and OTC, respectively). Although most DTCs are associated with favorable prognosis, certain histologic variants demonstrate more aggressive biological behavior. In particular, OTC and specific PTC variants, including tall-cell, columnar cell, diffuse sclerosing, solid/trabecular, and hobnail subtypes, are associated with higher rates of recurrence and increased risk of distant metastases (1). Beyond these variants, poorly differentiated thyroid carcinoma (PDTC) and differentiated high-grade thyroid carcinoma (DHGTC) represent biologically more aggressive entities within the spectrum of follicular-cell–derived thyroid malignancies. According to the current WHO classification, PDTC is recognized as a distinct tumor type intermediate between differentiated and anaplastic thyroid carcinoma, whereas DHGTC represents a high-grade form of differentiated thyroid carcinoma characterized by adverse histopathologic features and less favorable clinical outcomes (2,3).

Most patients with DTC achieve excellent outcomes following standard therapy, which includes thyroidectomy with or without radioactive iodine (RAI) treatment and risk-tailored thyroid-stimulating hormone (TSH) control, with 5-year survival rates exceeding 98% in both low- and intermediate-risk categories (4,5). However, approximately 5%–15% of cases present with radioiodine-refractory thyroid carcinoma (RAIR-TC), a condition defined by absent, lost, or heterogeneous RAI uptake, or disease progression despite adequate RAI. Prognosis of RAIR-TC cases declines substantially, with reported 10-year survival rates often below 20%–30% and median survival frequently in the range of 3–6 years in patients with progressive metastatic disease. Managing RAIR-TC remains a major clinical challenge, as systemic treatment options are limited to tyrosine kinase inhibitors, which are often associated with significant toxicity and variable efficacy (6,7). This heterogeneous disease spectrum may also encompass tumors with more dedifferentiated features, including PDTC and DHGTC, which are frequently characterized by impaired iodine avidity and more aggressive clinical course (8).

Within this continuum, a particularly difficult diagnostic entity is thyroglobulin-elevated negative iodine scintigraphy (TENIS) syndrome, characterized by persistently elevated serum thyroglobulin (Tg) levels despite negative diagnostic or post-therapy RAI scans. It represents a critical clinical dilemma: while rising Tg reliably suggests residual or recurrent disease, the inability to localize lesions restricts therapeutic intervention. Accurate lesion mapping is essential because identifying resectable or otherwise ablatable disease can substantially alter management and prognosis (9).

Current guidelines from the American Thyroid Association (ATA) (10) recommend FDG positron emission tomography/computed tomography (PET/CT) in this setting. As a hybrid molecular imaging modality, PET/CT provides both metabolic and anatomical information, facilitating whole-body tumor detection and staging. FDG PET/CT has shown diagnostic value in patients with elevated Tg and negative iodine scans, particularly for aggressive or dedifferentiated lesions. However, it has notable limitations. Uptake is not tumor-specific and may be increased in inflammatory, infectious, or physiological conditions, reducing specificity. Moreover, FDG PET/CT has reduced sensitivity for small-volume or slowly proliferating lesions and is often suboptimal for detecting pulmonary or cerebral metastases, where physiologic glucose uptake obscures tumor signal. These limitations underscore the need for novel radiotracers capable of improving detection sensitivity and diagnostic accuracy in this patient population (11–13).

One promising approach involves targeting the tumor microenvironment rather than tumor cells themselves. Fibroblast activation protein (FAP), a type II integral membrane serine protease highly expressed on cancer-associated fibroblasts but minimally present in normal tissues, has emerged as an attractive molecular target (14). Radiolabeled fibroblast activation protein inhibitors (FAPI) have been developed for FAPI PET/CT imaging, offering rapid blood clearance, low background activity, and high tumor-to-background contrast (13). Early oncologic studies demonstrated intense FAPI uptake in a variety of epithelial malignancies, including breast, pancreatic, colorectal, and lung cancers, reflecting the universal role of cancer-associated fibroblasts in tumor stroma formation and progression (15).

Initial experience in thyroid malignancies has been encouraging. A recent systematic review by Rizzo et al. highlighted the emerging role of FAPI PET/CT in both DTC and medullary thyroid carcinoma (MTC), showing consistently high detection rates for local recurrence and distant metastases, including lymph nodes, lung, bone, liver, and pleura, with superior contrast compared to FDG PET/CT in several studies (16). Individual reports have also underscored its value in RAIR-TC and TENIS cases. Fu et al. described a patient with TENIS in whom [68Ga]Ga-FAPI PET/CT identified metastatic disease not seen on FDG PET/CT (17). Similarly, Chen et al. demonstrated high detection rates and management impact in a prospective RAIR-TC cohort scanned with [^68^Ga]-DOTA-FAPI-04 PET/CT (18). These findings suggest that FAPI PET/CT may complement or, in selected cases, outperform FDG PET/CT, particularly in iodine-negative or FDG-non-avid disease.

Beyond diagnosis, FAPI’s tumor-specific uptake pattern offers a potential theranostic pathway. Because the same fibroblast activation protein can be targeted with therapeutic radioisotopes such as 177Lu or 225Ac, FAPI-based radioligand therapy (RLT) is under active investigation. Early-phase studies have reported favorable biodistribution, acceptable dosimetry, and preliminary evidence of antitumor activity across solid malignancies, providing a rationale for its future use in RAIR-TC—a population that currently lacks effective targeted radionuclide therapies (19).

The present systematic review was conducted to critically appraise and synthesize all available evidence focusing exclusively on RAIR-TC and TENIS syndrome. Specifically, we aimed to evaluate the diagnostic performance of FAPI PET/CT, compare its detection capability and lesion contrast with FDG PET/CT, and explore its potential complementary and theranostic roles in the management of iodine-negative DTC.

## 2 Methods

This review was reported according to the Preferred Reporting Items for Systematic Reviews and Meta-Analyses (PRISMA) guidelines (Supplementary Table 1) (20). No ethical approval or informed consent was required. Moreover, this systematic review was prospectively registered in the International Prospective Register of Systematic Reviews (21) (PROSPERO; registration number CRD420251123809). The record is publicly available at https://www.crd.york.ac.uk/PROSPERO/view/CRD420251123809.

### 2.1 Search strategy

We comprehensively and systematically searched three electronic databases (PubMed, Embase, and Scopus) from their inception to 6-August 2025. The search utilized the following terms: (“FAPI” OR “Fibroblast activation protein inhibitor” OR “FAP” OR “fibroblast activation protein” OR “68Ga-FAPI” OR “18F-FAPI”) AND (“PET” OR “PET/CT” OR “Positron Emission Tomography” [MeSH Terms]) AND (“Thyroid cancer”) AND (“TENIS” OR “TENIS syndrome” OR “thyroglobulin elevated negative iodine scintigraphy” OR “radioiodine-refractory” OR “RAIR”). The complete search strategy was adapted for each database and included controlled vocabulary and free-text terms related to FAPI tracers and thyroid cancer. Reference lists of eligible studies were also manually screened to identify additional relevant publications.

### 2.2 Eligibility criteria

This review included studies that evaluated the role of FAPI PET/CT in patients with RAIR-TC or TENIS syndrome. Case reports were included due to the limited number of available studies investigating FAPI PET/CT specifically in RAIR-TC and TENIS syndrome. Exclusion criteria comprised non-original research articles (e.g., review articles, systematic reviews, and conference or meeting abstracts), and studies conducted in non-human or *in vitro* settings. Studies combining RAIR-TC or TENIS patient groups with patients who had radioiodine-sensitive thyroid cancer without providing a separate analysis, were likewise excluded. Publications in languages other than English were also excluded.

### 2.3 Screening and data extraction

Two reviewers independently screened titles and abstracts using Rayyan (22). Following this, they independently performed a secondary screening by thoroughly reviewing the full text of the identified studies based on predetermined inclusion criteria. Subsequently, they independently extracted data from the included studies using a predesigned Microsoft Excel sheet. The extracted data included the title, year, authors, study location, study design, diagnosis subtype, sample size, age, gender and specific FAPI ligand. Key variables assessed included detection rates, lesion-to-background contrast, and comparison with FDG PET/CT or other imaging modalities. Semiquantitative PET measures, including median Maximum Standardized Uptake Value (SUVmax) and Tumor-to-Background Ratio (TBR) were also assessed if available.

### 2.4 Risk of bias and quality assessment

The risk of bias and quality assessment of the included studies were independently performed using the Quality Assessment of Diagnostic Accuracy Studies-2 (QUADAS-2) tool (23). This tool assesses potential sources of bias in four key domains: patient selection, index test, reference standard, and flow and timing. Each domain was judged as having a low, high, or unclear risk of bias based on signaling questions. Two independent reviewers performed the risk of bias assessment, with discrepancies resolved through discussion or consultation with a third reviewer when necessary. The overall quality of the studies was determined based on the degree of bias observed across domains. The evaluation was performed using Excel and the respective results were presented visually using robvis (24).

## 3 Results

### 3.1 Study selection

**FIGURE 1:**
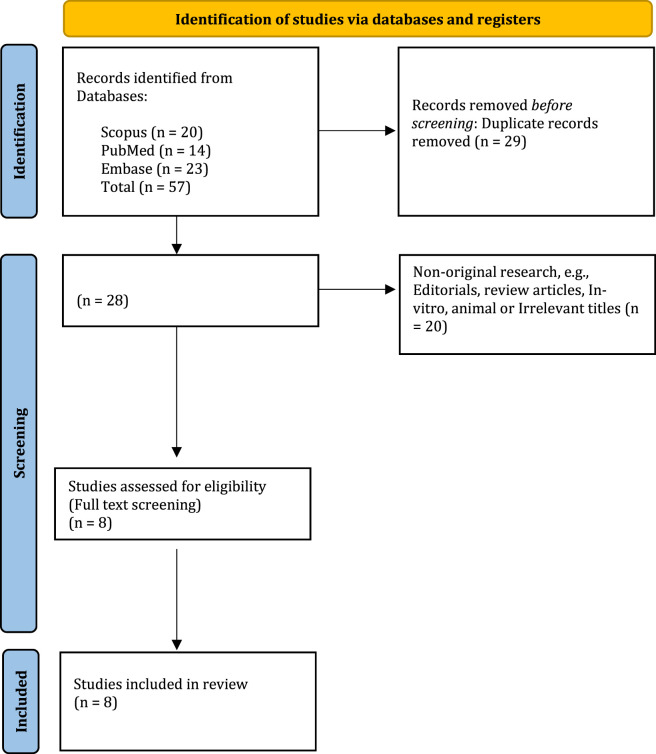
Illustrates the selection process using the PRISMA flow diagram.

The database search yielded a total of 57 records (Figure 1) (Scopus = 20, PubMed = 14, Embase = 23). After removal of 29 duplicates, 28 unique studies were retained for title and abstract screening. Of these, 20 records were excluded for being non-original research (e.g., review articles, editorials), *in vitro* or animal studies, or otherwise irrelevant based on title/abstract. The remaining 8 full-text articles were assessed for eligibility, and all of them met the inclusion criteria. Consequently, 8 studies were included in the final qualitative synthesis.

### 3.2 Characteristics of the included studies

This systematic review includes eight studies (17,18,25–30) investigated the diagnostic utility of FAPI PET/CT in patients with RAIR-TC or TENIS syndrome (Table 1), including three prospective studies, one retrospective study, and four case reports. The studies were conducted in China, India, Iran, and Cyprus, with a total of 167 patients. Several FAPI tracers were evaluated, including [^68^Ga]Ga-DOTA-FAPI-04, [^68^Ga]Ga-DOTA.SA.FAPi, [^68^Ga]Ga-FAPI-46, [^68^Ga]Ga-FAPI-RGD, and [^18^F]FAPI-74. Most studies included comparison with FDG PET/CT, while others used CT, MRI, histopathology, clinical follow-up, or thyroglobulin trends for lesion validation. Histologic subclassification was variably reported across studies; therefore, patients were categorized according to the disease definitions provided in the original publications.

**TABLE 1:** Summarizes the characteristics of the included studies.

Title	Location	Study type	No.	Gender	Age	Diagnosis	FAPI tracer	Comparison	Key diagnostic result
(25)	China	Case report (interesting image)	1	M	50	RAIR-TC	[^68^Ga]-FAPI	FDG PET/CT	Higher TBR than FDG; better detection of small pulmonary metastases
(17)	China	Case report (interesting image)	1	M	66	TENIS	[^68^Ga]-FAPI	CT	Intense uptake in laryngeal and pulmonary metastases
(18)	China	Prospective	24	7 M/17 F	49.6 ± 10.5	RAIR-TC	[^68^Ga]-DOTA-FAPI-04	CT, Tg	Patient-based detection rate 87.5% (21/24)
(27)	India	Retrospective	117	68 F/49 M	53.2 ± 11.7	RAIR-TC	[^68^Ga]-DOTA.SA.FAPi	FDG PET/CT	Higher detection than FDG for LN, liver, and brain metastases
(28)	Iran	Case report	1	F	46	TENIS	[^68^Ga]-FAPI	FDG PET/CT, DOTATATE	Only positive imaging modality; iliac bone metastasis detected
(26)	China	Case report (interesting image)	1	F	60	RAIR-TC	[^68^Ga]-FAPI-RGD	FDG PET/CT	Higher uptake and clearer margins than FDG for skull metastasis
(29)	Iran	Prospective	14	3 M/11 F	51.6 ± 12.2	RAIR-TC	[^68^Ga]-FAPI-46	FDG PET/CT	Similar detection to FDG; lower background uptake
(30)	Cyprus	Prospective	10	7 M/3 F	40–81	RAIR-TC	[^18^F]-FAPI-74	FDG PET/CT	FDG superior: Lesion detection 29/71 vs. 13/71 (p < 0.001)

### 3.3 Risk of bias and quality assessment

**FIGURE 2:**
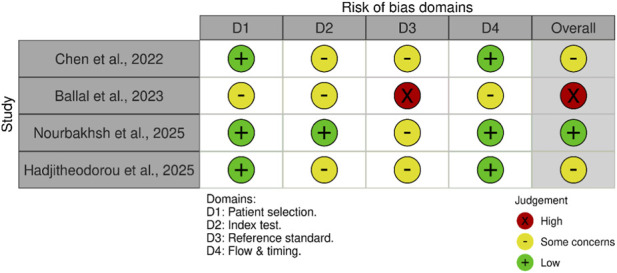
QUADAS-2, quality assessment of diagnostic accuracy studies.

QUADAS-2 was applied to the four cohort studies (Figure 2), including three prospective studies and one retrospective study. The four case reports were not formally assessed because single-patient descriptive reports do not provide the comparative diagnostic framework required for QUADAS-2 evaluation. Overall, patient selection and flow/timing were judged as low risk or low concern in most studies. The main methodological limitations involved the index test and reference standard domains, mainly because blinding of image interpretation was not consistently reported and lesion validation frequently relied on CT, clinical follow-up, thyroglobulin trends, or lesion growth rather than uniform histopathologic confirmation. The retrospective cohort by Ballal et al. (27) was judged as high risk in the reference standard domain because lesion verification was heterogeneous, including CT, clinical follow-up, and occasional histopathology, which may introduce verification or misclassification bias. Detailed domain-level justifications are provided in Supplementary Table 2.

### 3.4 Diagnostic performance and comparative findings of FAPI PET/CT

Overall, most studies reported higher tumor-to-background contrast with FAPI PET/CT compared with FDG PET/CT, although lesion detection rates varied depending on tracer type and tumor location.

Fu et al. (25) (2021) reported a case of a 50-year-old male with RAIR-TC previously treated with thyroidectomy and 5.5 GBq of radioiodine. Despite thyroglobulin elevation (132 ng/mL), FDG PET/CT identified only limited disease, while [^68^Ga]-Ga-FAPI PET/CT uncovered more extensive lung metastases with markedly higher uptake (SUVmax 12.9 vs. 6.1). The higher tumor-to-background ratio suggested that FAPI may be a promising alternative for detecting occult RAIR-TC lesions and for guiding FAP-targeted radionuclide therapy.

In another illustrative case from the same group (17), a 66-year-old man with TENIS syndrome was evaluated. Having received 3.7 GBq of RAI 7 years prior, he presented with a laryngeal mass and bilateral pulmonary nodules. [^68^Ga]-Ga-FAPI PET/CT demonstrated intense uptake in all known lesions and revealed an additional hilar focus missed on CT. This emphasized FAPI’s diagnostic utility in iodine-negative disease and its potential to guide management when FDG or CT are limited.

A prospective single-center study conducted in China by Chen et al. (18) (2022) included 24 patients with RAIR-TC. [^68^Ga]-Ga-DOTA-FAPI-04 PET/CT achieved an 87.5% detection rate (detected 21 out of 24 found by CT). SUVmax values correlated significantly with subsequent lesion growth (p = 0.047) but not with serum thyroglobulin. The mean SUVmax was 4.25 across lesions. This finding suggested that FAPI uptake may reflect tumor biology and aggressiveness more reliably than serum biomarkers, offering potential theranostic value.

The largest cohort to date was described by Ballal et al. (27) (2023), who retrospectively analyzed 117 patients with RAI-refractory follicular cell–derived thyroid cancer. Compared with FDG, [^68^Ga]-Ga-DOTA.SA.FAPi demonstrated superior detection of lymph nodes (95.4% vs. 86.6%), liver (100% vs. 81.3%), brain (100% vs. 39%), and bowel metastases, while pulmonary metastases were also more often detected with FAPI (81.7% vs. 64.6). Bone and pleural metastases were similarly visualized by both tracers. Quantitatively, FAPI uptake was significantly higher in brain and muscle lesions (SULpeak 13.9 vs. 6.7, and 9.6 vs. 5.6, respectively). The study concluded that FAPI outperformed FDG in metastatic sites and could be a powerful theranostic adjunct.

A case from Iran by Aghaee et al. (28) (2023) illustrated FAPI’s added value when other modalities failed. A 46-year-old woman with TENIS syndrome and a stimulated thyroglobulin of 499 ng/mL had negative FDG and DOTATATE PET/CT scans. However, [^68^Ga]-Ga-FAPI PET/CT detected a right iliac bone metastasis (SUVmax 2.9), later confirmed by MRI and pathology after surgical resection. This case highlighted the ability of FAPI to localize osseous disease overlooked by conventional tracers, facilitating curative-intent surgery and highlighting its potential as a theranostic guide.

In 2024, Chen et al. (26) reported the first use of [^68^Ga]-Ga-FAPI-RGD, a bispecific tracer targeting FAP and integrin αvβ3. A 60-year-old female with RAIR-TC and rising thyroglobulin (1948 → 2485 ng/mL) after two RAI treatments (7.4 GBq) presented with a skull metastasis. Both FDG and FAPI-RGD PET/CT detected the lesion, but FAPI-RGD provided sharper lesion boundaries and superior contrast due to lower brain background uptake (SUVmax 8.0, TBR 8.0 vs. FDG SUVmax 8.7, TBR 5.7). This suggested an added value of FAPI tracer, including potential for improved theranostics when radiolabeled with β- or α-emitters.

In Iran, Nourbakhsh et al. (29) (2025) performed a prospective head-to-head study of 14 patients with RAIR-TC (stimulated Tg > 10 ng/mL). Both FDG and [^68^Ga]-Ga-FAPI-46 PET/CT identified disease in 10/14 patients. FAPI demonstrated significantly lower physiologic background uptake (blood pool SUVmax 1.7 vs. 2.0; liver 1.25 vs. 2.65, p < 0.001). While overall lesion detection was similar, one patient with pulmonary metastases was FDG-negative (SUVmax 0.9) but FAPI-positive (SUVmax 3.8).

Lastly, Hadjitheodorou et al. (30) (2025) presented interim prospective data from Cyprus on 10 RAIR-TC patients scanned with [^18^F]-FAPI-74. FDG detected more lesions in three patients, and FAPI-74 missed recurrence in one. On a per-lesion basis, FDG significantly outperformed FAPI (29/71 vs. 13/71, p < 0.001), and all FAPI-positive lesions were also FDG-positive. Tumor-to-background ratios favored FDG (median TBRmax 3.05 vs. 1.09, p < 0.001). The authors concluded that FDG PET/CT remains the gold standard for RAIR-TC, but FAPI could serve as a complementary tool in FDG-negative cases and as a potential theranostic tool. Table 2 presents the comparative diagnostic and semiquantitative findings.

**TABLE 2:** Comparative diagnostic and semiquantitative findings of FAPI PET/CT in RAIR-TC and TENIS syndrome.

Study	Clinical setting	Tg level when reported	FAPI tracer	Comparator	Patient-based detection	Lesion-based/Site-based findings	Semiquantitative findings	Reference standard/Validation	Management impact
(25)	RAIR-TC case report	Tg 132 ng/mL	[^68^Ga]Ga-FAPI	FDG PET/CT	FAPI-positive; FDG-positive but less extensive	FAPI detected more extensive pulmonary metastatic disease than FDG	FAPI SUVmax 12.9 vs. FDG SUVmax 6.1; higher TBR with FAPI	Imaging correlation and clinical context	Suggested improved disease mapping and potential eligibility for FAP-targeted therapy
(17)	TENIS case report	Not clearly reported in manuscript text	[^68^Ga]Ga-FAPI	CT	FAPI-positive	FAPI showed intense uptake in laryngeal and pulmonary lesions and revealed an additional hilar focus missed on CT	NR	CT correlation and clinical context	Additional lesion detection; potential impact on staging/localization
(18)	Prospective RAIR-TC cohort	Tg assessed; correlation with FAPI uptake not significant	[^68^Ga]Ga-DOTA-FAPI-04	CT, Tg, clinical history	87.5% patient-based detection, 21/24	FAPI-positive lesions corresponded to clinically relevant RAIR-TC sites; lesion growth correlated with uptake	Mean SUVmax 4.25; SUVmax correlated with subsequent lesion growth, p = 0.047	Clinical history, CT, Tg, and follow-up	Potential prognostic/theranostic relevance; FAPI uptake may reflect lesion behavior
(27)	Retrospective RAIR follicular-cell–derived thyroid cancer cohort	NR	[^68^Ga]Ga-DOTA.SA.FAPi	FDG PET/CT	NR	Higher FAPI detection for lymph nodes, liver, brain, bowel, and lung; similar detection for bone and pleura	Brain lesions: FAPI SULpeak 13.9 vs. FDG 6.7; muscle lesions: FAPI SULpeak 9.6 vs. FDG 5.6	Imaging-based comparison and clinical validation; histopathology not uniformly available	Improved metastatic mapping; potential theranostic selection
(28)	TENIS case report	Stimulated Tg 499 ng/mL	[^68^Ga]Ga-FAPI	FDG PET/CT, DOTATATE PET/CT, MRI	FAPI-positive; FDG-negative; DOTATATE-negative	FAPI detected right iliac bone metastasis missed by FDG and DOTATATE	FAPI SUVmax 2.9	MRI and postsurgical pathology	Enabled lesion localization and surgical resection
(26)	RAIR-TC case report	Tg increased from 1948 to 2485 ng/mL	[^68^Ga]Ga-FAPI-RGD	FDG PET/CT	Both FAPI-RGD and FDG positive	Both detected skull metastasis; FAPI-RGD provided clearer lesion margins and lower brain background	FAPI-RGD SUVmax 8.0, TBR 8.0 vs. FDG SUVmax 8.7, TBR 5.7	Imaging correlation and clinical context	Suggested improved lesion delineation and theranostic potential
(29)	Prospective RAIR-TC cohort	Stimulated Tg > 10 ng/mL eligibility criterion	[^68^Ga]Ga-FAPI-46	FDG PET/CT	Both FAPI and FDG detected disease in 10/14 patients	Overall detection similar; one patient with pulmonary metastases was FDG-negative but FAPI-positive	Blood pool SUVmax 1.7 vs. 2.0; liver SUVmax 1.25 vs. 2.65; p < 0.001. FDG-negative/FAPI-positive lung lesion: FDG SUVmax 0.9 vs. FAPI SUVmax 3.8	Imaging comparison and follow-up	Possible upstaging in selected FDG-negative disease
(30)	Prospective RAIR-TC cohort	NR	[^18^F]FAPI-74	FDG PET/CT	FAPI-positive lesions were also FDG-positive	FDG detected more lesions than FAPI: 29/71 vs. 13/71, p < 0.001; FAPI missed recurrence in one patient	Median TBRmax favored FDG: 3.05 vs. 1.09, p < 0.001	Imaging comparison and clinical assessment	Did not support routine superiority of FAPI-74 over FDG; suggests tracer-dependent performance

Abbreviations: FDG, fluorodeoxyglucose; FAPI, fibroblast activation protein inhibitor; NR, not reported; RAIR-TC, radioiodine-refractory thyroid carcinoma; TENIS, thyroglobulin-elevated negative iodine scintigraphy; SUVmax, maximum standardized uptake value; SULpeak, peak standardized uptake normalized to lean body mass; TBR, tumor-to-background ratio; Tg, thyroglobulin.

## 4 Discussion

This systematic review synthesizes the currently available evidence on the diagnostic utility of FAPI PET/CT in RAIR-TC and TENIS syndrome, two clinically challenging settings in thyroid cancer imaging. The available literature remains limited, comprising eight studies with 167 patients, including three prospective studies, one retrospective cohort, and four case reports. Overall, FAPI PET/CT demonstrated encouraging diagnostic performance in selected clinical contexts, particularly for lesions with limited FDG conspicuity or in anatomical sites where physiologic FDG uptake may impair lesion detection. Nevertheless, comparative performance varied across tracers, lesion sites, patient populations, and study designs. Accordingly, the current evidence supports a potential complementary role for FAPI PET/CT rather than establishing its routine diagnostic superiority over FDG PET/CT.

The biological rationale for FAPI PET/CT in RAIR-TC and TENIS syndrome is compelling. FDG PET/CT reflects increased glucose metabolism and remains the established molecular imaging modality in patients with elevated thyroglobulin and negative radioiodine imaging, particularly in the context of dedifferentiated or clinically aggressive disease (10–12). However, FDG PET/CT may be limited by physiologic background activity, inflammatory uptake, and reduced sensitivity for small-volume or slowly proliferating lesions. FAPI PET/CT, by contrast, targets fibroblast activation protein expression within cancer-associated fibroblasts in the tumor microenvironment (14,15). This mechanism may partly explain the favorable lesion-to-background contrast reported in several included studies, particularly for metastases in the brain, liver, bowel, and lymph nodes (18,27). However, FAP expression is not restricted to malignant tissue, and FAPI uptake should therefore be interpreted within the broader clinical, biochemical, and anatomical imaging context (31,32).

Among gallium-labeled FAPI tracers, several studies reported favorable diagnostic findings. In the largest available cohort, Ballal et al. showed higher detection rates with [^68^Ga]Ga-DOTA.SA.FAPi than with FDG PET/CT for selected metastatic sites, including lymph nodes, liver, brain, bowel, and lung lesions, while detection of bone and pleural metastases was broadly comparable between tracers (27). Chen et al. reported that [^68^Ga]Ga-DOTA-FAPI-04 uptake was significantly associated with subsequent lesion growth, suggesting that FAPI avidity may reflect biologically relevant tumor–stromal activity (18). In addition, case reports in RAIR-TC and TENIS syndrome illustrated the potential of FAPI PET/CT to identify clinically relevant lesions that were inconspicuous or negative on FDG PET/CT, DOTATATE PET/CT, or CT (17,25,26,28). Collectively, these findings suggest that FAPI PET/CT may provide incremental diagnostic information in selected patients.

Importantly, however, the comparative evidence is not uniform. Nourbakhsh et al. reported similar patient-level disease detection between [^68^Ga]Ga-FAPI-46 PET/CT and FDG PET/CT, although FAPI PET/CT demonstrated lower physiologic background uptake and identified pulmonary metastatic disease in one FDG-negative patient (29). Conversely, Hadjitheodorou et al. reported inferior lesion detection and lower tumor-to-background ratios with [^18^F]FAPI-74 compared with FDG PET/CT, with all FAPI-positive lesions also detected by FDG (30). These discrepant findings underscore the influence of tracer pharmacokinetics, ligand structure, acquisition protocols, lesion biology, and patient selection on diagnostic performance. They also indicate that evidence from one FAPI ligand should not be generalized to all FAPI-based tracers.

The interpretation of FAPI PET/CT in RAIR-TC and TENIS syndrome must also account for the substantial clinical and biological heterogeneity of these disease states. Patients may differ in histologic subtype, degree of dedifferentiation, metastatic distribution, iodine avidity, FDG avidity, thyroglobulin level, prior treatment exposure, tumor burden, and tempo of disease progression (1–7). Such heterogeneity limits the generalizability of findings from small single-center cohorts and isolated case reports. Therefore, future investigations should stratify diagnostic performance according to clinically relevant variables, including histology, lesion site, iodine avidity, FDG status, thyroglobulin burden, prior therapy, and FAPI ligand.

Tracer heterogeneity represents another important source of variability. Most available data involve gallium-labeled tracers, including [^68^Ga]Ga-DOTA-FAPI-04, [^68^Ga]Ga-FAPI-46, and [^68^Ga]Ga-DOTA.SA.FAPi (18,27,29). Although fluorine-labeled compounds may offer logistical advantages through a longer physical half-life and potential centralized production, the available [^18^F]FAPI-74 data in RAIR-TC did not demonstrate superiority over FDG PET/CT (30). Bispecific tracers such as [^68^Ga]Ga-FAPI-RGD may also have distinct biodistribution and lesion-contrast characteristics (26). These observations reinforce the need for tracer-specific validation rather than assuming class-wide diagnostic equivalence among FAPI agents.

A further limitation of FAPI PET/CT is the potential for non-malignant uptake. FAP expression may occur in benign fibro-inflammatory, reparative, or fibrotic processes, including post-surgical and post-radiation changes, healing fractures, inflammatory lesions, atherosclerosis, pancreatitis, and chronic fibrotic lung disease (31). This issue is particularly relevant in thyroid cancer, where post-treatment cervical changes, pulmonary abnormalities, and osseous lesions may complicate image interpretation. Reports of non-malignant FAPI uptake in TENIS syndrome further emphasize that FAPI positivity should not be equated with malignancy (32). Correlation with CT or MRI morphology, thyroglobulin dynamics, lesion evolution, and histopathology when feasible remains essential to reduce false-positive interpretation.

The theranostic implications of FAPI imaging are promising but remain investigational. FAPI PET/CT may help identify lesions with sufficient tracer uptake for consideration of FAP-targeted radioligand therapy, a potentially relevant strategy in RAIR-TC where therapeutic options after loss of iodine avidity are limited (19,33,34). Early clinical experience with FAPI-directed radioligand therapy in metastatic RAIR thyroid cancer supports feasibility and provides a rationale for further evaluation (33). However, diagnostic uptake alone does not establish therapeutic efficacy. Optimal patient selection, ligand choice, radionuclide selection, dosimetry, treatment sequencing, response assessment, and long-term toxicity remain insufficiently defined. Thus, FAPI-based theranostics in RAIR-TC should currently be regarded as an investigational strategy requiring prospective validation.

Several limitations should be emphasized. First, the number of available studies remains small, and half of the included publications were case reports, limiting generalizability. Second, heterogeneity was evident in study design, patient selection, tracer type, imaging protocol, comparator modality, and reference validation. Third, histopathologic confirmation was not uniformly available, and most studies relied on composite validation using CT, MRI, clinical follow-up, thyroglobulin trends, or lesion growth, introducing potential verification and misclassification bias. Fourth, blinding of image interpretation was inconsistently reported. Finally, the limited number of studies and substantial methodological heterogeneity precluded meta-analysis and prevented robust subgroup analyses by tracer, lesion site, histology, or FDG status.

Future prospective multicenter studies should incorporate standardized imaging protocols, harmonized interpretation criteria, predefined patient-based and lesion-based endpoints, and robust reference validation. Head-to-head comparisons with FDG PET/CT should report lesion-site-specific performance, semiquantitative parameters, thyroglobulin levels, iodine and FDG avidity status, and management impact. Outcome-oriented endpoints, including treatment modification, eligibility for FAPI-targeted therapy, progression-free survival, and patient-centered outcomes, may further clarify whether FAPI PET/CT provides clinically meaningful benefit beyond lesion detection.

In summary, FAPI PET/CT represents a promising adjunctive imaging modality in RAIR-TC and TENIS syndrome, particularly in selected patients with negative, equivocal, or diagnostically limited conventional imaging. However, the current evidence remains preliminary, heterogeneous, and insufficient to establish routine diagnostic superiority over FDG PET/CT. At present, its most defensible role is as a complementary imaging tool in carefully selected clinical scenarios and as a potential biomarker for future FAPI-directed theranostic strategies.

## 5 Conclusion

FAPI PET/CT demonstrates promising but preliminary diagnostic utility in patients with RAIR-TC and TENIS syndrome. Current evidence suggests that FAPI PET/CT may provide complementary value in selected clinical scenarios, particularly when iodine imaging or FDG PET/CT is negative, equivocal, or limited by physiologic background uptake. However, comparative performance varies across FAPI tracers, lesion sites, patient populations, and study designs, and the available evidence remains insufficient to establish routine diagnostic superiority over FDG PET/CT. The potential theranostic role of FAPI imaging is clinically relevant, but remains investigational and requires prospective validation. Larger multicenter studies with standardized imaging protocols, robust reference standards, lesion-based and patient-based endpoints, and outcome-based assessment are needed to define the clinical role of FAPI PET/CT in RAIR-TC and TENIS syndrome.

## Data Availability

The data extracted and analyzed during this systematic review were derived from previously published studies. The extracted data supporting the findings of this review are available from the corresponding author upon reasonable request.
